# Clinical and serological characterization of acute pleuropericarditis suggests an autoinflammatory pathogenesis and highlights risk factors for recurrent attacks

**DOI:** 10.1007/s00392-024-02390-w

**Published:** 2024-02-15

**Authors:** Dorothee Kaudewitz, Lukas John, Jan Meis, Norbert Frey, Hanns-Martin Lorenz, Florian Leuschner, Norbert Blank

**Affiliations:** 1 Department of Internal Medicine V, Hematology, Oncology and Rheumatology, Im Neuenheimer Feld 410, 69120 Heidelberg, Germany; 2 Institute of Medical Biometry, University of Heidelberg, Heidelberg, Germany; 3 Department of Internal Medicine III (Cardiology, Angiology, and Pneumology), Heidelberg University Hospital, Im Neuenheimer Feld 410, 69120 Heidelberg, Germany; 4 DZHK (German Center for Cardiovascular Research), Partner Site Heidelberg/Mannheim, 69120 Heidelberg, Germany

**Keywords:** Pleuritis, Pericarditis, Antinuclear antibodies, Serum amyloid A, Autoinflammation

## Abstract

**Purpose:**

We describe the manifestations and course of patients with pleuropericarditis (PP). Serum parameters were analyzed to evaluate the contribution of autoimmune and autoinflammatory mechanisms to PP pathogenesis. Finally, we outline risk factors for recurrent PP attacks.

**Methods:**

Electronic medical records of the University Hospital Heidelberg were screened for PP diagnosis between the years 2009 and 2021. A total of 164 patients were detected and compared to patients suffering from systemic lupus erythematosus (SLE)-associated PP. Follow-up data were collected until January 2023.

**Results:**

In 57.3% of a total of 164 PP cases, no trigger was identified (idiopathic PP). The clinical manifestations were similar in subgroups with different triggers (idiopathic, post-cardiac injury and post-infectious). None of the patients in the idiopathic-PP (i-PP) group fulfilled the diagnostic criteria of an autoimmune disease and the i-PP group could be clearly discriminated by clinical, epidemiological and serological means from the control cohort of SLE-associated PP. After a median follow-up of 1048 days, the majority of PP patients (72.7%) had at least one PP relapse. Univariate analyses showed that CRP, SAA (serum amyloid A), troponin T, NT-BNP and post-cardiac injury were negatively correlated, while the presence of fever and an idiopathic trigger were positively correlated with recurrence of PP. Multivariate analyses showed that fever, an idiopathic trigger and low SAA values were risk factors for PP recurrence.

**Conclusion:**

This study highlights that most cases of PP are idiopathic and PP cases with various triggers have an identical clinical phenotype. Our data suggest that the clinical, epidemiological and serological characteristics of idiopathic PP considerably differ from patients with PP caused by autoimmune disease like SLE. We further demonstrate that PP has a high risk of recurrence and identify factors associated with this risk, allowing for a targeted secondary prophylaxis.

**Graphical Abstract:**

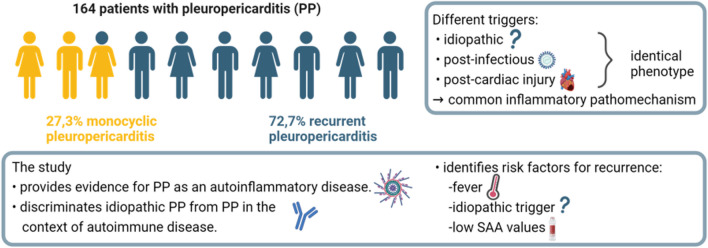

**Supplementary Information:**

The online version contains supplementary material available at 10.1007/s00392-024-02390-w.

## Introduction

Acute pleuropericarditis (PP) is the most common inflammatory heart disorder and accounts for about 5% of all emergency department admissions for acute chest pain [1, 2]. Although acute PP usually is a mild and self-limiting disease, about 30% of patients experience complications, e.g. cardiac tamponade or recurrent PP [1, 3]. Recurrent episodes can profoundly impact the quality of life, leading to repeated hospital admissions and adverse effects related to long-term pharmacological therapy [1, 4–7].

While various triggers for PP have been described including viral infections, mycobacterial infections, vaccinations with mRNA vaccines against SARS-CoV-2, or cardiac tissue injury [8–14], the exact mechanism of inflammation remains elusive. An autoimmune aetiology was hypothesized because of the association of PP with various systemic rheumatic diseases like systemic lupus erythematosus (SLE), rheumatoid arthritis or vasculitis [15]. On the other hand, PP is also a typical feature of autoinflammatory syndromes like familial Mediterranean fever, TNF receptor-associated periodic syndrome, systemic juvenile idiopathic arthritis or adult onset Still’s disease [15]. In addition, mouse models suggested a role for inflammasome activation and IL-1β. Genetic analyses showed an increased frequency of rare MEFV (MEditerranean FeVer) mutations in PP patients, again suggesting an association of autoinflammatory mechanisms in the pathogenesis of PP [3, 16, 17].

Importantly, the risk factors for recurrence of PP remain unclear. Patients at risk for recurrent PP may require a more intense treatment of the first attack and more frequent follow-up visits. Previous studies claimed that an increased neutrophil/lymphocyte ratio (NLR) and higher age are risk factors for recurrent PP [4].

In order to better understand PP pathogenesis and identify risk factors for recurrence, we performed a retrospective analysis of PP patients treated at the University Hospital Heidelberg over the course of more than 10 years. Clinical phenotypes were compared according to the presence or absence of PP trigger events. The contribution of autoinflammatory versus autoimmune mechanisms to PP pathogenesis was assessed by comparing clinical, epidemiological and serological parameters in idiopathic PP with SLE-associated PP.

## Methods

The electronic medical records of the University Hospital Heidelberg were screened for PP diagnosis between the years 2009 and 2021. In order to avoid wrong diagnoses, only patients who were additionally discussed with a rheumatologist were included in the further analysis. We further included all patients who were identified by interdisciplinary counseling between cardiologists and rheumatologists between 2009 and 2021 at our institution. Patients with known autoinflammatory diseases or with effusions due to heart, kidney or liver failure or effusions classified as transudates were excluded from this analysis. Follow-up data were collected until January 2023. This study was performed in line with the principles of the Declaration of Helsinki and was approved by the University of Heidelberg Ethics committee (S-550/2023).

Pericarditis was diagnosed according to the European Society of Cardiology guidelines when at least two of the following criteria were met: typical chest pain, pericardial friction rub, characteristic electrocardiogram changes or pericardial effusion [18]. Pericardial and pleural effusions were classified according to the Light criteria [19, 20].

Patients were assigned to three different categories of triggers: Patients with a current infection or a history of an infection within 4 weeks before the PP diagnosis were considered having post-infectious PP (pi-PP). Patients who had a myocardial ischemia or required invasive cardiovascular interventions and developed PP within 4 weeks after the intervention were diagnosed with post-cardiac injury PP (pc-PP). PP without associated infections, vaccinations, rheumatic diseases, myocardial ischemia or previous cardiac diseases was considered idiopathic PP (i-PP).

The clinical course was assigned as monocyclic, polycyclic or chronic PP according to the ESC recommendations [18]. Polycyclic PP was defined as the occurrence of symptoms and signs of PP after a period of complete resolution of symptoms. Chronic PP was defined as the persistence of symptoms for at least 3 months regardless of anti-inflammatory treatment. Due to the small number, chronic courses were excluded from further analyses.

To address the question of autoimmunity vs. autoinflammation, i-PP patients were compared to a control group of 15 patients with SLE and autoimmune polyserositis treated at our institution between the years 2009 and 2021. The diagnosis of systemic lupus erythematosus (SLE) was consistent with the recently published classification criteria by EULAR and ACR [21]. These criteria use an ANA (anti-nuclear antibodies) titre of ≥ 1:80 on Hep-2 cells as an entry criterion and subsequently evaluate clinical symptoms including pericarditis, pleural and pericardial effusions as signs of polyserositis.

All patients underwent routine diagnostic procedures, including clinical history, vital signs, electrocardiography, echocardiography, pleura sonography and routine laboratory analysis. The presence of fever was defined as a temperature of at least 38.0 °C. In a select set of patients with clinical indication, additional data was collected such as cardiac MRI (68 patients) or aspiration of effusion (35 patients). An exact overview on the number of patients with these clinical characteristics is shown in Suppl. Table 1. The median follow-up time for patients was 1048 days as of January 31, 2023. A total of 32 patients were lost to follow-up and were excluded from the analysis of recurrence.

Additionally, we registered the treatments received to evaluate how frequently treatment beyond the standard medication of colchicine and prednisolone is needed. Due to the retrospective nature of the study and a lack of randomization, we did not perform further analyses of the effectiveness of the treatments.

### Statistical analyses

Numeric variables were summarized using medians, 25% and 75% quantiles, and categorical variables were reported via absolute and relative frequencies. For numeric variables, Kruskal–Wallis-one-way analyses of variance and two-sided Wilcoxon rank sum tests were used to derive *p*-values for descriptive comparisons. The latter test was also used as post hoc test in case of statistical significance in the Kruskal–Wallis test. Categorical variables were compared by chi-squared and Fisher-Boschloo tests [22]. The threshold for statistical significance was defined as 0.05 for *p*-values derived from two-sided tests. As the nature of our study was exploratory, no corrections for multiple testing were performed. Those values meeting statistical significance in the univariate model (*p* < 0.05) were included as candidate variables for a multivariable logistic regression model to investigate whether the observed effects persist when adjusting for other factors. Model selection was performed based on the Akaike information criterion. Cutoff points were determined using the cutpointr package maximizing the sum of sensitivity and specificity. All statistical analyses were conducted using R software version 4.2.2 [23]. In the further analysis of the data, patients with chronic pleuropericarditis were excluded as the statistical analysis was limited due to small patient numbers in this group.

## Results

### Patient characteristics

A total of 164 patients were included in the analysis. These patients were treated for monocyclic, recurrent or chronic PP in our tertiary single centre University Hospital Heidelberg between the years 2009 and 2021. The median follow-up time was 1048 days as of January 31, 2023. An overview of patients’ characteristics is presented in Table 1. Most patients presented with both pleural and pericardial effusions (*n* = 106 (64.6%)). Only a small percentage showed an isolated pericardial (*n* = 29 (17.7%)) or pleural (*n* = 24 (14.6%)) effusion. Twenty-seven patients required pericardiocentesis and 17 patients required pleurocentesis. According to the Light criteria, these effusions were classified as exudates, fitting to the concept of PP as an inflammatory disease. Five patients (3.0%) presented with the typical clinical symptoms of PP but were diagnosed with pleuropericarditis sicca; i.e. no pleural or pericardial effusion was detected.

**Table 1:** Demographic characteristics of the study population

	All patients *n* = 164	Idiopathic *n* = 94 (57.3)	Post-cardiac injury *n* = 49 (29.9)	Post-Infectious *n* = 21 (12.8)	*p*-value
Females; *n* (%)	84 (51.2)	47 (50.0)	30 (61.2)	7 (33.3)	0.095
Age; years	53 [38–68]	52 [37–68]	60 [44–73]	54 [36–62]	0.210
Clinical signs					
Fever (> 38.0 °C); *n* (%)	78 (47.3)	46 (48.9)	20 (40.8)	12 (57.1)	0.419
Pleural effusion	130 (73.9)	75 (79.8)	42 (85.7)	13 (61.9)	0.078
Pericardial effusion	135 (82.3)	80 (85.1)	39 (79.6)	16 (76.2)	0.524
Myocarditis on MRI	18 (11.0)	13 (13.8)	2 (4.1)	3 (14.3)	0.182
ST-Elevation	17 (10.4)	13 (13.8)	2 (4.1)	2 (9.5)	0.191
Low voltage	11 (6.7)	5 (5.3)	4 (8.2)	2 (9.5)	0.697
Smoker	51 (31.1)	28 (29.8)	16 (32.7)	7 (33.3)	0.914
Biomarkers					
CRP max; mg/l	149 [77–200]	151 [68–203]	132 [75–171]	162 [90–261]	0.433
CRP mg/l	42 [7–134]	14 [5–137]	64 [22–124]	65 [15–168]	0.099
SAA mg/l	65 [9–491]	25 [7–392]*	187 [65–521]*	126 [36–568]	0.038
Procalcitonin ng/ml	0.1 [0.1–0.2]	0.1 [0.1–0.2]	0.1 [0.1–0.2]	0.2 [0.1–2.8]	0.051
Troponin T pg/ml	11 [6–29]	8 [4–21]**	18 [9–71]**	15 [5–340]	0.007
Ferritin µg/l	235 [82–560]	193 [73–516] *	268 [105–435] *	601 [311–821]*	0.011
NT-BNP ng/l	497 [148–1667]	360 [129–1082]	968 [269–5175]	889 [86–3622]	0.060
Leukocyte count/nl	10.9 [8.0–13.8]	10.8 [8.2–13.8]	10.2 [7.5–12.3]	14.8 [9.8–18.4]	0.137
Neutrophil count/nl	7.9 [5.9–10.7]	8.0 [6.1–10.7]	6.9 [5.1–9.8]	10.3 [7.4–13.8]	0.413
Lymphocyte count/nl	1.4 [1.1–2.0]	1.4 [1.2–1.9]	1.3 [1.1–2.0]	1.7 [0.8–2.4]	0.888
N/L-ratio	6.4 [3.9–8.6]	6.3 [4.6–8.2]	7.3 [3.4–8.4]	6.8 [3.2–13.9]	0.904
ANA titre	1:80 [0–320]	1:80 [0–320]	1:80 [0–320]	1:80 [40–160]	1.000
Course					
Monocyclic; *n* (%)	33 (20.1)	12 (12.8)**	17 (28.6)**	4 (19.0)	0.008
Polycyclic; *n* (%)	88 (53.7)	58 (61.7)	21 (42.9)	9 (42.9)	0.057
Chronic; *n* (%)	11 (6.7)	7 (7.4)	3 (4.1)	1 (4.8)	0.889
Lost to follow-up; *n*	32 (19.5)	17 (18.1)	8 (24.5)	7 (33.3)	0.224
Treatment					
Colchicine	126 (76.8)	73 (77.7)	39 (79.6)	14 (66.7)	0.481
Prednisolone	123 (75.0)	69 (73.4)	39 (79.6)	15 (71.4)	0.663
NSAR	67 (40.9)	45 (47.9)	15 (30.6)	7 (33.3)	0.104
Anakinra	20 (12.2)	14 (14.9)	4 (8.2)	2 (9.5)	0.467

The group of patients with idiopathic, post-cardiac injury, and post-infectious PP were compared among each other. The data are presented as number (percent) or as median [Interquartile range]. CRP max indicates the maximum CRP value during the first episode.* refers to *p*-value < 0.05, ** refers to *p*-value < 0.01 and marks the individual groups with significant differences between each other. *ANA* anti-nuclear antibody, *N/L* neutrophil/leukocyte, *NSAR* non-steroidal anti-inflammatory drugs

Myocarditis was detected in 11.0% (18/68) of cardiac MRIs. In the group of patients with the diagnosis of myocarditis in the MRI, 64.7% (11/17) of patients with evaluable troponin had elevated troponin levels (median 173 pg/ml [6–428]) and 56.3% (9/16) of patients with evaluable NT-BNP had elevated NT-BNP levels (median 161 ng/l [57–1360]). We did not observe any cases of chronic myocarditis in our cohort.

The troponin levels were generally low, with a median value of 10.7 ng/l. During the first episode of PP, we saw serological signs of acute inflammation with elevated CRP levels in 78.8% and elevated SAA (serum amyloid A) levels in 71.2%, with a median CRP value of 148.7 mg/l (upper limit of normal: 5) and a median SAA value of 64.6 mg/l (upper limit of normal: 6.4).

Out of 132 patients with follow-up, the majority (88 patients) experienced at least one relapse after initial complete resolution of pleural or pericardial effusion, while only few cases of chronic effusion (8.3%) were seen in our cohort. Patients with a chronic course showed only mildly elevated inflammation markers (median CRP 14.9 mg/l, median SAA 4.3 mg/l), thus maybe explaining the low response to anti-inflammatory therapy.

Patients were treated according to the ESC guidelines [18]. Unless there were contraindications, all patients were treated with NSAIDs and colchicine (0.5 mg twice daily). Colchicine therapy was continued for at least 6 months. If severe manifestations were present or if this treatment was not sufficient, prednisolone (0.5mg/kg/day) with continuous tapering was added to the therapy. For most patients in our cohort, treatment with colchicine or prednisolone was sufficient to achieve a symptom-free condition. Additional medication such as azathioprine was only given in 25 out of 164 patients. Only 20 patients (12.2%) received further treatment with IL-1-inhibitors such as anakinra.

### Underlying autoimmunity is not detected in patients with idiopathic PP

Some authors have suggested that PP is the symptom of an underlying autoimmune disease, while others have proposed autoinflammation as the underlying pathomechanism.

**Fig. 1:**
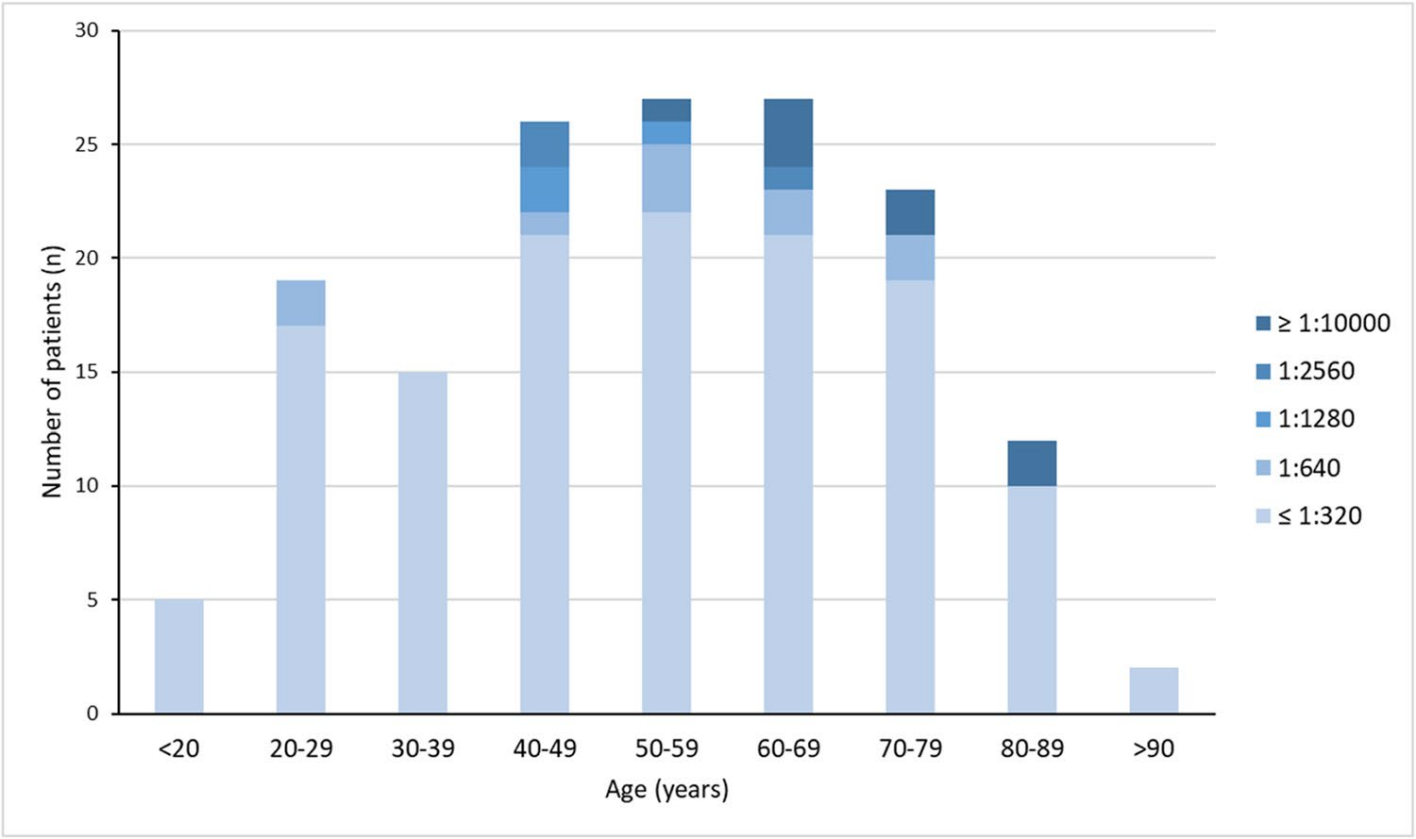
Age dependent distribution of ANA titres in the PP cohort. Different shades of blue indicate the height of the ANA titres. ANA, anti-nuclear antibody

To further investigate these hypotheses, we screened our patients for clinical and serological parameters typical for autoimmunity. None of the patients in our PP cohort fulfilled the ACR criteria for the classification of SLE. We searched for anti-nuclear antibodies (ANA) that could hint at an underlying autoimmune process. In 85% of patients in our cohort, we did not find relevantly elevated ANA levels (> 1:320) and only six patients showed specificity for extracted nuclear antigens. High levels of ANA (> 1:2560) were exclusively seen in older patients above the age of 50 years (Fig. 1) and unlike in autoimmune diseases, women were not overrepresented in this group (51.2% female vs. 48.8% male).

To further discriminate the PP cohort from patients with PP caused by autoimmune disease, we compared our idiopathic PP group with a cohort of SLE patients with symptomatic polyserositis (SLE-PP) (Table 2). SLE has a clear predilection for women between the age of 15 and 44 years [24]. Elevated ANA titres are characteristic and an ANA titre of ≥ 1:80 is used as an entry criterion in the classification criteria [21]. In our cohort, we saw that the ANA values were significantly higher (*p* < 3.2 × 10^−8^) in the SLE-PP group, where an ANA value above 1:320 with a homogenous fluorescence pattern was detected in all patients. The average age was significantly lower in the SLE-PP group (37.0 years SLE vs. 52.0 years i-PP, *p* = 0.021). Unlike in the i-PP group, where the gender distribution was equal, 86.7% of patients in the SLE-PP group were female. We saw that the highest CRP value during the first episode (median 77.5 mg/l SLE-PP vs. 150.5 mg/l i-PP) was on average lower in the SLE group, though no statistically significant difference was detected (*p* = 0.189).

**Table 2:** SLE cohort

	i-PP cohort *n* = 164	SLE + PP controls *n* = 15	*p*-value
Females; *n* (%)	47 (50.0)	13 (86.7)	0.019*
Age at 1st PP; years; median [IQR]	52 [37–68]	37 [26–50]	0.021*
CRP maximum mg/l	151 [68–203]	78 [13–185]	0.189
CRP at visit-1 mg/l	14 [5–137]	12 [3–26]	0.464
SAA at visit-1 mg/l	25 [7–392]	109 [68–188]	0.267
ANA titre; median [IQR]	1:80 [0–320]	1:10,000 [2560–30,000]	< 0.0001***

Comparison of the i-PP (idiopathic pleuropericarditis) cohort with a cohort of SLE patients with polyserositis, CRP max indicates the maximum CRP value during the first episode. The data are presented as number (percent) or as median [interquartile range]. * refers to *p*-value < 0.05, ** refers to *p*-value < 0.01, *** refers to *p*-value < 0.001. *ANA* anti-nuclear antibody

Taken together, the clinical, epidemiological and serological characteristics of our cohort of PP patients considerably differ from patients with PP caused by autoimmune disease like SLE.

### Different triggers converge on a common pathway

To determine the influence of the trigger on the manifestations and course of the disease, we assigned our cohort to three groups: idiopathic PP (i-PP), post-cardiac injury PP (pc-PP) and post-infectious PP (pi-PP).

Out of 164 patients, 49 patients (29.9%) developed PP after cardiac injury, 21 patients (12.8%) after an infection and 94 patients (57.3%) developed PP without any defined trigger. Between the three triggers, we did not see significant differences regarding the clinical phenotype (Table 1).

However, we saw significant differences regarding the serological alterations between the groups. As expected, the troponin T levels were highest in the post-cardiac injury group (median 18.0 pg/ml vs. i-PP 8.0 pg/ml, Wilcoxon *p* = 0.002). The SAA levels were lowest in the group with an idiopathic trigger (i-PP median 25.2 pg/ml vs. pc-PP 187.0 mg/l, Wilcoxon *p* = 0.019) and the highest ferritin levels were seen in the post-infectious group (median 600.5 µg/l vs. pc-PP 268.0 µg/l, Wilcoxon *p* = 0.031; and i-PP 193.0 µg/l, Wilcoxon *p* = 0.003) with procalcitonin also showing a trend for higher levels in this subgroup (Wilcoxon *p* = 0.051).

In summary, while different triggers of PP lead to differences in specific laboratory values, no differences with regard to clinical presentation or general routine laboratory markers are observed.

### An idiopathic trigger is strongly associated with the risk of recurrence

Recurrent episodes of PP have a profound impact on patients’ quality of life and morbidity. Therefore, patients with a high likelihood of recurrence may benefit from stricter surveillance and earlier and/or more intense treatment. Out of 121 patients with follow-up (median follow-up 1048 days, excluding chronic PP), the majority (72.7%) experienced at least one relapse after initial complete resolution of pleural or pericardial effusion. In this group, 47.7% of patients experienced further episodes of PP after the first relapse. Notably, the trigger was strongly associated with the risk of recurrence (Table 3). 82.9% of patients in the idiopathic group experienced recurrence (*p* = 0.003) compared to only 55.3% of patients in the post-cardiac injury group (*p* = 0.006) and 69.2% in the post-infectious group (*p* = 0.684).

**Table 3:** Risk factors for recurrence

	Monocyclic PP *n* = 33 (27.3%)	Polycyclic PP *n* = 88 (72.7%)	Kruskal–Wallis *p*-value
Females	16 (48.5)	44 (50.0)	1.000
Age at first attack, years	54 [42–65]	49 [36–66]	0.391
Follow-up, days	1093 [247–1271]	1048 [415–1962]	0.781
Clinical signs			
Fever (> 38.0 °C); *n* (%)	11 (33.3)	53 (60.2)	0.011*
Pleural effusion; *n* (%)	26 (78.8)	74 (84.1)	0.542
Pericardial effusion; *n* (%)	25 (75.8)	73 (83.0)	0.416
Myocarditis on MRI; *n* (%)	7 (21.2)	9 (10.2)	0.112
ST-elevation; *n* (%)	6 (18.2)	10 (11.4)	0.332
Low voltage; *n* (%)	2 (6.1)	5 (5.7)	1.000
Smoker; *n* (%)	9 (27.3)	27 (30.7)	0.800
Biomarker			
CRP max mg/l	160 [124–225]	150 [79–200]	0.087
CRP mg/l	96 [31–169]	20 [4–126]	0.002**
SAA mg/l	203 [70–998]	26 [8–353]	0.007**
Procalcitonin ng/ml	0.1 [0.1–0.2]	0.1 [0.1–0.2]	0.479
Troponin T pg/ml	18 [9–228]	11 [5–26]	0.032*
Ferritin µg/l	389 [216–470]	176 [77–537]	0.077
NT-BNP ng/l	1137 [439–2528]	307 [114–1245]	0.015*
Leukocyte count /nl	10.4 [8.1–13.9]	11.7 [9.8–15.7]	0.178
Neutrophil count /nl	9.8 [6.7–13.7]	7.4 [5.1–10.6]	0.160
Lymphocyte count /nl	1.4 [1.2–1.7]	1.5 [1.0–2.1]	0.484
N/L-ratio	7.6 [6.3–10.4]	6.3 [3.9–7.6]	0.131
ANA titre	1:160 [40–320]	1:80 [0–320]	0.720
Trigger			
Idiopathic	12 (17.1)	58 (82.9)	0.003**
Post-infectious	4 (30.8)	9 (69.2)	0.684
Post-cardiac injury	17 (44.7)	21 (55.3)	0.006**

Comparison of patients with a monocyclic and a recurrent course. The data are presented as number (percent) or as median [interquartile range]. 32 patients that were lost to follow-up and 11 patients with chronic PP were not included in the table. CRP max indicates the maximum CRP value during the first episode. * refers to *p*-value < 0.05, ** refers to *p*-value < 0.01. *ANA* anti-nuclear antibody, *N/L* neutrophil/leukocyte, *NSAR* non-steroidal anti-inflammatory drugs

### Low SAA values and fever are risk factors for recurrence

We additionally performed a univariate analysis (Table 3) to identify clinical risk factors as well as serological biomarkers to anticipate the risk of recurrence. We identified significant differences between the patients with a monocyclic course (m-PP) versus a recurrent course (r-PP). The inflammatory markers at presentation were significantly lower in patients who experienced recurrence (CRP at presentation (median r-PP 20.2 mg/l vs. m-PP 95.7 mg/l, *p* = 0.002) and SAA (median r-PP 26.4 mg/l vs. m-PP 202.5 mg/l, *p* = 0.007)). Regarding cardiac parameters, we saw significantly lower values in the polycyclic group (troponin T median r-PP 10.7 pg/ml vs. m-PP 17.5 pg/ml, *p* = 0.032 and NT-BNP median r-PP 307.0 ng/l vs. m-PP 1137.0 ng/l, *p* = 0.015).

In addition, the number of patients presenting with fever was significantly higher in the polycyclic group (r-PP 53/88 (60.2%) vs. m-PP 11/33 (33.3%), *p* = 0.011).

### Multivariate analysis identifies fever as the strongest predictor for recurrence

As a next step, we performed a multivariate analysis with the factors that showed significance in the univariate analysis and selected the best fitting model using the Akaike information criterion as a fitting criterion (Table 4). The best model contained SAA, TnT, NT-BNP, the presence of fever and idiopathic genesis as the most important predictors. Fever was the strongest predictor for recurrence (OR 42.2*, p* = 0.003). An idiopathic trigger was significantly associated with an increased risk of recurrence (OR 9.8, *p* = 0.035), while high SAA values negatively correlated with recurrence (OR 0.7/100 units, *p* = 0.017). In order to better interpret SAA levels, we performed a cut point analysis which identified the level of 63.9 mg/l as the cut point maximizing the sum of sensitivity (0.61) and specificity (0.81) in predicting a polycyclic course (see supplementary Fig. 1). However, prospective studies are required to better assess the suitability of SAA as a biomarker for relapse. The correlation of NT-BNP and TnT values (OR 1.03/100 units, *p* = 0.202 and OR 0.737/100 units, *p* = 0.086) was less profound. However, this correlation might be due to the confounding effect caused by the higher NT-BNP and TnT values in the post-cardiac injury group compared to the other groups.

**Table 4:** Multivariate analysis of risk factors for recurrence

	Monocyclic	Polycyclic	Odds ratio	95% CI	*p*-value
Serum amyloid A mg/l	203 [70–998]	26 [8–353]	0.760	[0.586–0.941]	0.017 *
NT-BNP ng/l	1137 [439–2528]	307 [114–1245]	1.027	[1.003–1.091]	0.202
TnT pg/ml	18 [9–228]	11 [5–26]	0.737	[0.500–0.973]	0.086
Idiopathic	12 (36.4)	58 (65.9)	9.794	[1.405–115.473]	0.035 *
Fever	11 (33.3)	53 (60.2)	42.207	[5.085–932.770]	0.003 **

Multivariate analysis of risk factors for recurrence of PP. Parameters which were significantly associated with recurrence in the univariate analysis were analyzed using logistic regression. The final model was selected based on Akaike information criterion. Only patients with completely documented values were included (*n* = 50). Effect size is presented as odds ratio with a 95% confidence interval (95% CI), where odds ratios larger than one signal an increase in the estimated adjusted odds for recurrence. SAA, NT-BNP, and TnT are given as OR per 100 units change. The data are presented as number (percent) or as median [interquartile range]. * refers to *p*-value < 0.05, ** refers to *p*-value < 0.01

In summary, our data indicate that higher levels of inflammatory markers, such as CRP and SAA, are associated with a decreased risk of recurrence, as are cardiac damage markers such as troponin T and NT-BNP. The most important risk factors for recurrent PP according to the multivariate analysis are fever at diagnosis, an idiopathic trigger and low SAA values.

## Discussion

In this study, we present the analysis of a large cohort of 164 patients with PP who were treated at the University Hospital Heidelberg over the course of more than 10 years. We investigate whether idiopathic PP could be differentiated from PP in the context of autoimmune disease. When we compared patients with SLE-associated PP with idiopathic PP, we found that the clinical, epidemiological and serological characteristics considerably differ from patients with PP caused by autoimmune disease like SLE. We further observed that various trigger factors (post-cardiac injury, infectious, idiopathic) result in a similar phenotype. However, trigger factors are relevant for the risk of recurrent PP. We show that fever at PP diagnosis, an idiopathic trigger for PP and low SAA are risk factors for recurrent PP.

Although PP is relatively common, the exact pathogenesis is not well understood. An autoimmune mechanism provoked by different triggers has been proposed by some authors. It has been suggested that viral infections may stimulate autoimmune responses by molecular mimicry, and after cardiac injury, the release of cardiac autoantigens may activate T and B lymphocytes [25, 26]. Additionally, PP can be a complication of autoimmune disorders. In SLE patients, pericarditis can occur in up to 50% of cases, although often asymptomatically [26]. In our analysis, we found that autoantibodies in patients’ sera of our PP cohort were significantly different compared to patients with SLE-associated PP. In patients with SLE-associated PP, we detected high ANA titres with a homogeneous fluorescence pattern. Patients with idiopathic PP had either negative ANA or significantly lower ANA titres with an in most cases speckled fluorescence pattern and ENA were negative. None of the patients fulfilled the ACR criteria for the diagnosis of SLE. On the other hand, previous analyses have shown that rare deleterious MEFV variants were more prevalent in PP when compared with ancestry-matched controls [16, 17], pointing towards an autoinflammatory origin of the disease. PP patients usually respond well to colchicine and anti-IL1-targeted medication which is suggestive for an activation of inflammasomes and the role of IL-1 in the pathogenesis of PP [27]. The efficacy of IL-1 antagonists especially anakinra and rilonacept has been shown in large clinical trials. Therefore, this specific treatment should be strongly considered in patients with recurrent PP [28, 29]. Our results show that PP has many features in common with an autoinflammatory disease. The equal gender distribution, the occurrence at various ages, the elevation of CRP and SAA, the absence of specific ANA titres and the efficacy of colchicine and IL-1 inhibition suggest an autoinflammatory, rather than an autoimmune background. Thus, PP appears to be a new manifestation of autoinflammation and should be treated accordingly.

The majority of patients developed PP without any obvious trigger and thus were diagnosed with idiopathic PP. Post-cardiac injury PP and post-infectious PP were less common. We did not see significant differences between the clinical phenotypes resulting from different triggers consistent with previous reports [30, 31]. This suggests that autonomous autoinflammation, tissue damage and viral infections at the end trigger a common inflammatory pathway employing the activation of inflammasomes and the activation of IL-1β. Our data show that a distinction between the triggers is relevant for the prognosis as flare of PP are more frequent in idiopathic PP.

In our study, we further highlight a high risk of recurrence and outline risk factors for recurrence. We observed recurrent PP in 72.7% in our cohort, which is higher than previous estimates, such as the 30% estimate risk of relapse in the 2015 ESC guideline [18, 31]. The discrepancy could be explained by the fact that more severe cases might be overrepresented at the tertiary care setting at the University Hospital Heidelberg. The use of an increased neutrophil/lymphocyte ratio (NLR) and higher age was suggested previously to indicate a risk for PP recurrence [4, 32]. However, in our cohort, we could not confirm neither an association with age or with the NLR nor the absolute leukocyte count in the blood. Another report showed post-cardiac injury PP to be negatively associated with recurrence, which is consistent with our observations [33]. A multivariate analysis of our data showed that fever at diagnosis was the strongest risk factor for recurrence, followed by an idiopathic trigger and low SAA values. A possible interpretation could be that infections or cardiac damage might be transient triggers and the proinflammatory drivers might disappear in the course of the disease. Furthermore, a strong inflammatory reaction could also induce anti-inflammatory mechanisms which contribute to terminate a PP attack. However, an autoinflammatory predisposition might provide chronic or recurrent stimuli for a persistent autoinflammatory reaction. Further investigations are required to prove these hypotheses.

This study has limitations due to the single-centre cohort, the retrospective analyses and non-standardized assessment of laboratory parameters. The retrospective analysis of treatment response limited conclusions about the treatment efficacy. In addition, the risk factors identified in our cohort may not apply to patients in countries where infections like tuberculosis are more prevalent and common differential diagnoses for PP. As we only included patients who were discussed with a rheumatologist, there might be a selection bias towards more severe PP cases. Additionally, more severe cases might be overrepresented at the tertiary care setting. As this is a retrospective analysis, no randomization to different treatment groups was performed. Therefore, patients with more severe manifestations that did not resolve with colchicine alone were more likely to receive additional therapy. We did not include the different therapies into the multivariate model since this question has to be addressed in a prospective randomized trial.

## Conclusions

In conclusion, this study provides evidence that various triggers for PP lead to an identical phenotype implying a common inflammatory pathomechanism. It supports the concept of PP as an autoinflammatory disease and discriminates idiopathic PP from SLE-associated PP. Our analysis shows that the majority of PP patients will experience flares, and that fever, an idiopathic trigger and low SAA values are the most relevant risk factors for recurrence of PP.

## Supplementary Information

Below is the link to the electronic supplementary material.

Supplementary file1 (DOCX 221 KB)

## Data Availability

The datasets generated during and/or analyzed during the current study are available from the corresponding author on reasonable request.

## Abbreviations

ACR: American College of Rheumatology
ANA: Anti-nuclear antibody
CRP: C-reactive protein
ENA: Extractable nuclear antigens
EULAR: European League Against Rheumatism
N/L-ratio: Neutrophil/leukocyte-ratio
NSAR: Non-steroidal anti-inflammatory drugs
NT-BNP: N-terminal-brain natriuretic peptide
MRI: Magnetic Resonance Imaging
PP: Pleuropericarditis
SAA: Serum amyloid A
SLE: Systemic lupus erythematosus
