# Supplementary material for: Clinical and serological characterization of acute pleuropericarditis suggests an autoinflammatory pathogenesis and highlights risk factors for recurrent attacks
Source: Clin Res Cardiol. 2024 Feb 15;114(9):1109–17. doi: 10.1007/s00392-024-02390-w (PMC12408741; doi:10.1007/s00392-024-02390-w)
Supplement: Supplementary file 1 — Supplementary file1 (DOCX 221 KB) [file 392_2024_2390_MOESM1_ESM.docx]

### Supplementary Information:

**Supplementary table 1. Number of registered values in the study population**

|  | **All patients**  **n=164** | **Idiopathic**  **n=94** | **Post cardiac injury n=49** | **Post-Infectious n=21** |
| --- | --- | --- | --- | --- |
| Gender | 164 | 94 | 49 | 21 |
| Age | 164 | 94 | 49 | 21 |
| **Clinical signs** |  |  |  |  |
| Fever | 164 | 94 | 49 | 21 |
| Pleural effusion | 164 | 94 | 49 | 21 |
| Pericardial effusion | 164 | 94 | 49 | 21 |
| Cardiac MRI | 68 | 49 | 11 | 8 |
| ECG | 164 | 94 | 49 | 21 |
| Smoking | 164 | 94 | 49 | 21 |
| **Biomarkers** |  |  |  |  |
| CRP max | 162 | 92 | 49 | 21 |
| CRP | 151 | 87 | 45 | 19 |
| SAA | 88 | 53 | 25 | 10 |
| Procalcitonin | 96 | 53 | 31 | 12 |
| Troponin T | 132 | 77 | 37 | 18 |
| Ferritin | 119 | 70 | 35 | 14 |
| NT-BNP | 127 | 79 | 33 | 15 |
| Leukocyte count | 107 | 64 | 31 | 12 |
| Neutrophil count | 57 | 35 | 15 | 7 |
| Lymphocyte count | 57 | 35 | 15 | 7 |
| N/L-ratio | 57 | 35 | 15 | 7 |
| ANA titer | 156 | 91 | 46 | 19 |
| **Course** | 164 | 94 | 49 | 21 |
| **Treatment** | 164 | 94 | 49 | 21 |

Number of patients with registered values for each variable compared to the total number of 164 patients. ANA (anti-nuclear antibody), N/L neutrophil/leukocyte).

**Supplementary figure 1. SAA cutoff determination**


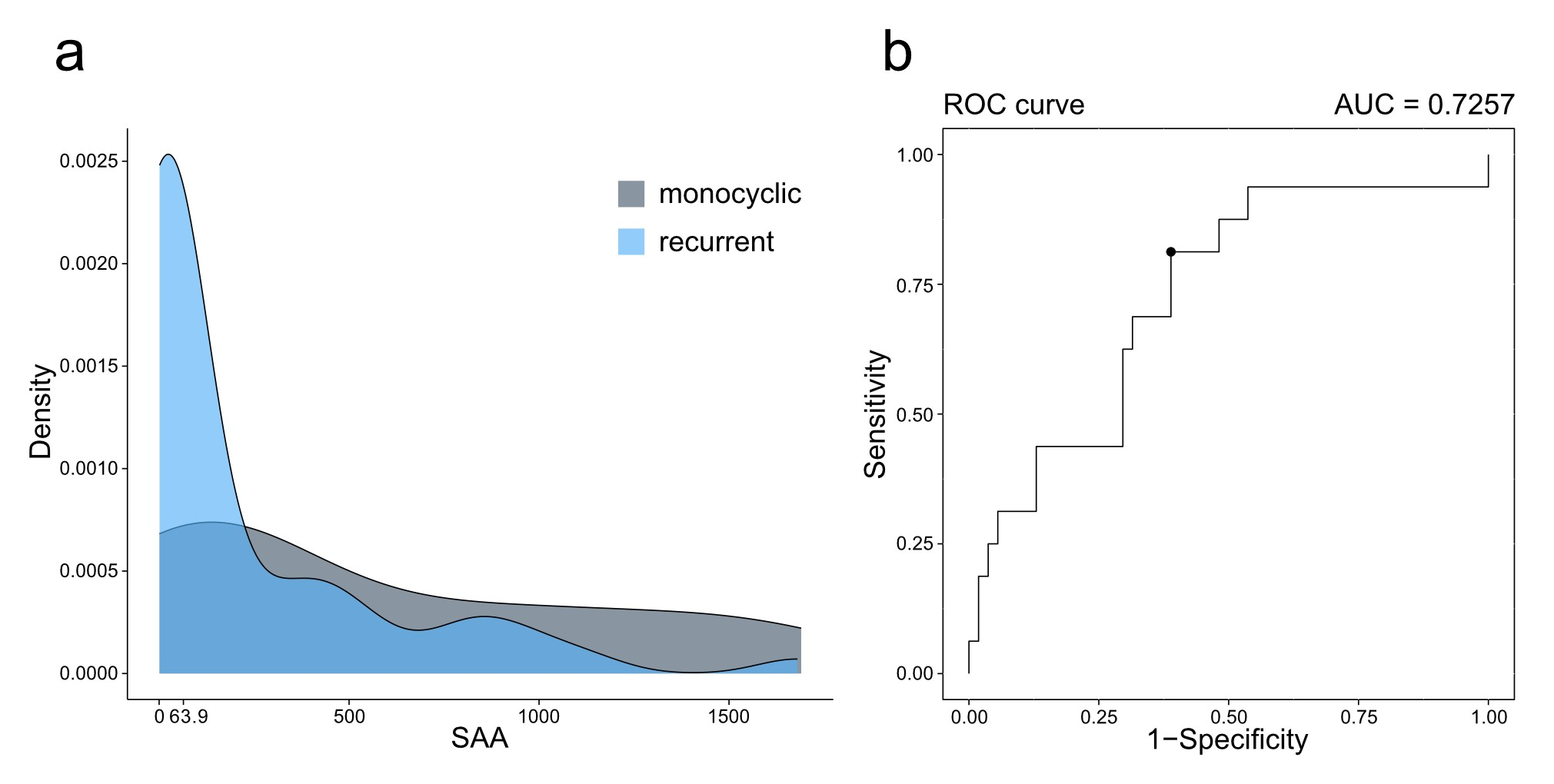


Suppl. Figure 1: SAA cutoff determination. (a) Density distribution of SAA values for patients with monocyclic (dark grey) and recurrent pleuropericarditis (light blue). Values are given in mg/l b) ROC-curve for SAA-cutoff determination. The AUC is 0.7257, the cutoff of 63.9 mg/l is denoted as a black dot.
